# Clinical and molecular profile of *Klebsiella pneumoniae* bloodstream infections in a national referral hospital in the Philippines

**DOI:** 10.1016/j.ijregi.2026.101039

**Published:** 2026-10-15

**Authors:** Christian Francisco, Angelo dela Tonga, Stessi Marie Geganzo, Joana Marie Gantuangco, Jonnel Poblete, Ia Marie Cruz, Bryan Santiago, Janet S. Lee, Tatsunori Oyabu, Masahiro Suzuki, Sohei Harada, Yohei Doi

**Affiliations:** 1 National Institutes of Health-University of the Philippines Manila, 623 Pedro Gil St. Ermita, Manila, 1000, Philippines; 2 Philippine General Hospital, Taft Ave, Ermita, Manila, 1000, Philippines; 3 Washington University in St. Louis, 660 S. Euclid Ave, St. Louis, MO, 63110, USA; 4 Fujita Health University, 1-98 Dengakugakubo, Kutsukake-cho, Toyoake, Aichi, 470-1192, Japan; 5 Toho University, 5 Chome 21-16 Omorinisihi, Ota City, Tokyo, 143-8540, Japan; 6 University of Pittsburgh, 4200 Fifth Ave., Pittsburgh, PA, 15260, USA

**Keywords:** *Klebsiella pneumoniae*, Antimicrobial resistance, Hypervirulence, Philippines

## Abstract

**Objectives:**

*Klebsiella pneumoniae* (Kp) bloodstream infections (BSIs) are a major concern in the Philippines, but local clinical and molecular epidemiologic data are limited.

**Methods:**

We conducted a cross-sectional study at a government hospital in Manila from April 2023 to January 2024, analyzing clinical data with whole genome sequencing of 39 cases.

**Results:**

Most patients were male (mean age 53 years) and had diabetes or malignancy. The in-hospital mortality rate was 38.5%. Extended-spectrum β-lactamase and carbapenemase genes were identified in 41.0% and 33.3% of isolates, respectively, predominantly *bla*_CTX-M-15_, *bla*_NDM_, and *bla*_KPC_. The most frequent sequence type was ST437, followed by ST23, ST101, and ST17. Hypervirulence genes (*rmpA/A2, iuc*, and *iro*) were detected in 25.6% of isolates, defining 10 hypervirulent Kp (hvKp) strains, including one carbapenem-resistant hvKp strain carrying *bla*_NDM-7_. Compared with classic Kp infections, hvKp infections resulted in higher rates of septic shock and infection-attributable mortality despite having lower multidrug resistance.

**Conclusion:**

These findings underscore the high mortality risk from Kp BSIs, driven by antimicrobial resistance and hypervirulence. The detection of hvKp and carbapenem-resistant hvKp is testament to the global spread of this critical priority pathogen.

## Highlights

• Detection of carbapenem-resistant hypervirulent strain denotes an emerging threat.
• High mortality is driven by antimicrobial resistance and hypervirulence.
• Sustained national genomic surveillance is needed to monitor spread.

## Introduction

The World Health Organization has identified *Klebsiella pneumoniae* (Kp) as a critical priority pathogen due to rising rates of antimicrobial resistance (AMR) and the limited treatment options available against multidrug-resistant (MDR) strains [1]. Kp infections are broadly classified into classic Kp (cKp) and hypervirulent Kp (hvKp), which can cause disseminated infections in otherwise healthy individuals [2]. Although urinary tract infections, cholangitis, and hospital-acquired pneumonia are the major infections caused by cKp, hvKp typically causes more fulminant infections, such as liver abscess, community-acquired necrotizing pneumonia, and bloodstream infections (BSIs) accompanying disseminated lesions, reflecting its ability to cause invasive disease [2]. In recent years, the global spread of MDR cKp and hvKp has reinforced Kp’s position on the World Health Organization’s research agenda for AMR in human health [3].

Kp accounts for approximately 10% of nosocomial infections worldwide [4]. This burden is compounded by high global prevalence rates of extended-spectrum β-lactamase (ESBL)–producing strains (32.7%) [5] and carbapenem-resistant (CR) strains (28.7%) [6]. In the Philippines, the 2024 Antimicrobial Resistance Surveillance Program (ARSP) of the Department of Health reported Kp as one of the three most frequently isolated pathogens from respiratory specimens, wound aspirates, urine, cerebrospinal fluid, and blood. More than half of Kp infections were community-acquired (54.5%), with phenotypic resistance to third-generation cephalosporins and carbapenems at 30.9% and 16.4%, respectively [7]. Despite this surveillance program, genomic data on Kp in the local setting remain scarce.

Kp pathogenicity is mediated by multiple virulence factors, including the capsule, lipopolysaccharides, siderophores, and fimbriae, which facilitate colonization, immune evasion, and tissue invasion [2]. A distinct pathotype, hvKp, is strongly associated with severe disseminated infections among healthy individuals [2]. In a study from India, mortality from hvKp infections was markedly higher than from cKp (87.5 vs 35.7%, *P* < 0.001) despite hvKp having lower MDR rates (44.4% vs 76.6%, *P* < 0.001) [8]. However, differentiating cKp from hvKp remains challenging due to the absence of a standardized definition [9] and the lack of reliable diagnostic assays for virulence-associated factors [10]. Operationally, hvKp is defined by the presence of *rmpA/A2, iro*, and *iuc* genes, with some studies also including *peg-344* [9,10]. Yet, the prevalence of these virulence genes in human Kp isolates in the Philippines has been minimally studied.

Of growing concern is the emergence of CR-hvKp in various health care centers [11]. This convergence of resistance and virulence occurs when plasmids carrying AMR and virulence genes are exchanged between CR Kp and hvKp or when cKp acquires hybrid plasmids harboring both genes [11]. The emergence of such strains poses a major threat to clinical outcomes and public health.

Given these challenges, understanding the clinical and molecular epidemiology of Kp is essential to guide treatment, inform infection control measures, and mitigate the impact of AMR and hypervirulence on the health care system and economy. This study aimed to describe the demographic characteristics, clinical features, and molecular profiles of Kp BSIs at the national referral hospital in Manila, Philippines.

## Methods

### Study design and setting

This report describes patients with serial Kp BSI who presented to the Philippine General Hospital, a tertiary government hospital in Manila, Philippines between April 2023 and January 2024. The report is part of an ongoing observational cohort study of patients with Kp BSI at hospitals in the Philippines and Japan.

The study protocol was approved by the University of the Philippines Manila Research Ethics Board (UPMREB) (protocol code 2023-0210-01). Informed consent was obtained from patients. In the case a patient was unable to provide consent due to the clinical condition, consent was sought from the nearest kin. A separate consent was obtained for biobanking and further testing of bacterial isolates. For patients who died before the release of the Kp identification report, UPMREB waived the requirement for consent.

### Study population

We recruited 60 consecutive adult patients with presumptive Kp BSI identified through an automated blood culture system. Preliminary identification of isolates was performed using VITEK MS (bioMérieux, Marcy-l'Etoile, France). Only patients who provided consent or were granted consent waivers by UPMREB were included. Patients with Kp infections from other body sites without concomitant BSI were excluded.

### Clinical data collection

Demographic, clinical, and laboratory data were obtained from electronic patient databases and medical records from the time of index admission until discharge or death. Variables collected included age, sex, comorbidities, infection source, intensive care unit (ICU) admission, ventilator use, presence of indwelling devices, surgical history, Pitt bacteremia score [12], severe sepsis, septic shock, source control procedures, and persistence of BSI. Data were recorded using a study-specific case report form and stored in a database developed with Research Electronic Data Capture software (Vanderbilt University, Nashville, TN, USA) hosted by Fujita Health University, Japan.

### Kp isolation, identification, and antibiotic susceptibility testing

Blood culture bottles were processed using BACT/ALERT VIRTUO (bioMérieux, Marcy-l'Etoile, France). Gram staining was performed, followed by subculture on blood agar plates that were incubated at 35°C for 8 hours. Presumptive Kp colonies were identified with VITEK MS (bioMérieux, Marcy-l'Etoile, France). Antibiotic susceptibility testing was performed with VITEK 2 XL (bioMérieux). Additional antimicrobial susceptibility testing (AST) for aztreonam, ceftazidime, levofloxacin, and tobramycin was carried out by disk diffusion in accordance with Clinical and Laboratory Standards Institute guidelines. The 2024 with Clinical and Laboratory Standards Institute break points were used to interpret the susceptibility of Kp to antimicrobial agents [13].

### Whole genome sequencing and bioinformatics analysis

Genomic deoxyribonucleic acid (DNA) was extracted using the QIAamp DNA Mini Kit (Qiagen, Germany), and concentration and purity were determined with a NanoDrop spectrophotometer (Thermo Fisher Scientific, Waltham, MA, USA). Extracted DNA samples were sent to Fujita Health University, Japan for sequencing. Library preparation was performed, and whole genome sequencing (WGS) was carried out on an Illumina NextSeq 2000 platform.

Raw reads were quality-checked using fastp v0.23.2 (OpenGene) and aligned with minimap2 v2.24 (Li H, Bioinformatics). Reference-based assemblies were generated with St. Petersburg Genome Assembler (SPAdes ) v3.15.4 (St. Petersburg State University), and the quality of the assemblies was assessed using Quality Assessment Tool for Genome Assemblies (QUAST) v5.2.0 (Center for Algorithmic Biotechnology, St. Petersburg State University). Species identification, AMR genes, virulence genes, sequence types (STs), and K and O antigen serotypes were determined using Kleborate v3.0.9 (University of Melbourne). AMR genotyping was performed using Kleborate’s curated version of the Comprehensive Antibiotic Resistance Database (CARD) (v4.0.1, May 2025 snapshot, McMaster University). STs were compared against the PubMLST Pasteur scheme (April 2024 snapshot), Institut Pasteur, whereas capsular (K) and lipopolysaccharide (O) surface antigens were predicted via the built-in Kaptive v3 databases (University of Melbourne). All analyses were conducted via a command-line interface.

### Operational definition of terms

Sepsis and severe sepsis were defined according to the 2001 International Sepsis Definitions [14]. HvKp was defined using the biomarker diagnostic method requiring the presence of all four clusters of virulence genes, which include *rmpA, rmpA2, iro*, and *iuc* [9]. Hospital-acquired infections were defined as infections that developed more than 48 hours after admission to a health care facility, and the remainder were defined as community-acquired infections [15]. Appropriate antibiotic use was defined as initiation of an antibiotic regimen (empiric and definitive treatment) against Kp BSI isolates that exhibited confirmed *in vitro* susceptibility and reliable tissue penetration and was supported by robust clinical data demonstrating superior efficacy, safety, and low propensity to drive further resistance [16]. Adequate source control was defined as the timely use of physical, intervention-based measures to eliminate the focus of infection and stop microbial spread.

### Statistical analysis

Clinical and demographic data were summarized using descriptive statistics. Categorical variables were presented as frequencies, and continuous variables were presented as means or medians, depending on the data distribution. Comparisons of categorical variables were performed using the chi-square test. Normally distributed continuous data were analyzed using Student’s *t*-test, whereas non-normally distributed data were compared using the Mann–Whitney U test. Descriptive statistics were also used for AST profiles and for the presence of AMR and virulence genes. Statistical analyses were performed with STATA v18 (Stata Corp, College Station, TX, USA). A Wellmap was generated using BioRender (Ontario, Canada). A phylogenetic tree and heat map were constructed using R v4.3.1 (R Foundation for Statistical Computing, Vienna, Austria).

## Results

### Study cohort

Of the 60 blood culture isolates initially collected from unique patients, 39 were confirmed as *K. pneumoniae sensu stricto* by WGS after the exclusion of 21 isolates: 11 due to contamination with other organisms (three mixed-species contamination [severe genome size inflation and fragmented assembly], four significant DNA carryover [high genome size inflation], four same genus/strain contamination [moderate overage]) and 10 identified as other members of the *K. pneumoniae* species complex (five as *Klebsiella quasipneumoniae* [one subspecies *quasipneumoniae* and four subspecies *similipneumoniae*], one as *Klebsiella africana*, one as *Klebsiella variicola*, and three as *Klebsiella* sp. without further speciation) (Figure 1).

**Figure 1:**
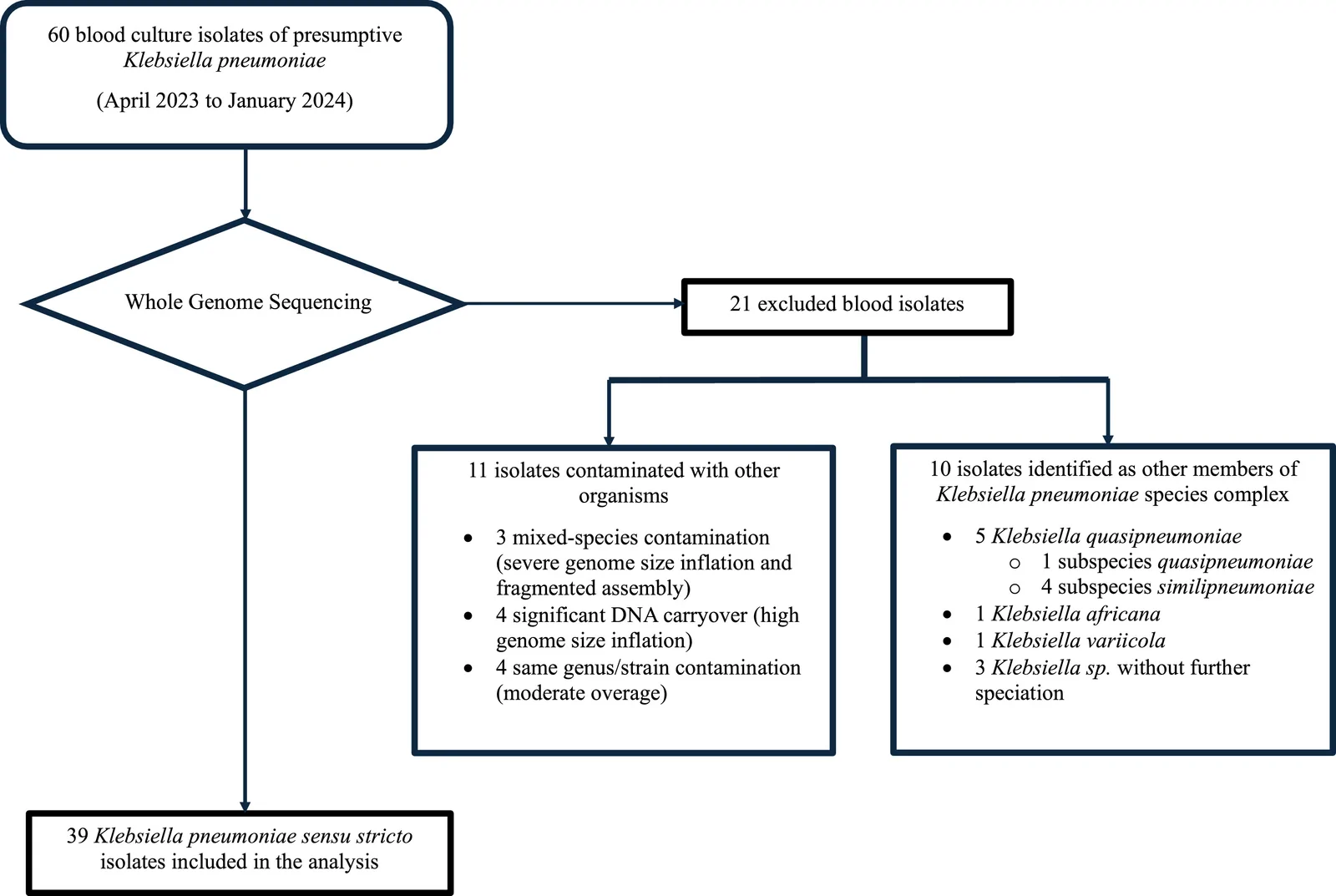
Flowchart of *Klebsiella pneumoniae* isolate selection, inclusion, and exclusion in the study. DNA, deoxyribonucleic acid.

### Clinical epidemiology

Among the 39 patients with Kp BSI, 21 (53.8%) were male, with a mean age of 53.8 years (SD 17.7). The most common comorbidity was diabetes mellitus (19 of 39; 48.7%), followed by solid tumor malignancies (12 of 39; 30.8%). Half of the infections were community-acquired (20 of 39; 51.3%). At BSI onset, four patients were already in the ICU and 12 were on mechanical ventilation.

Collectively, intra-abdominal infections were the most common focus of infection (14 of 39; 35.9%), including intra-abdominal abscesses or secondary peritonitis (seven of 39; 17.9%), cholangitis (six of 39; 15.4%), and spontaneous bacterial peritonitis (one of 39; 2.5%). This was followed by pneumonia (nine of 39; 23.1%), intravascular infections (eight of 39; 20.5%), and urinary tract infections (seven of 39; 17.9%). The mean Pitt bacteremia score at the time of the index blood culture was 3.3 (SD 3.7). Within the first week after BSI diagnosis, six additional patients required ICU admission, 24 developed severe sepsis, and 14 progressed to septic shock. The most common empiric antibiotic regimen included meropenem monotherapy (nine of 39; 23.1%), followed by meropenem/amikacin (four of 39; 10.2%) and meropenem/polymyxin B (four of 39; 10.2%) combination therapy. Ceftriaxone monotherapy was the most used definitive antibiotic regimen (six of 39; 15.4%), followed by ceftazidime-avibactam monotherapy (five of 39; 12.8%) and meropenem/polymyxin B combination therapy (five of 39; 12.8%) (Supplementary Table 1). A greater proportion of those who survived had appropriate empiric and definitive antibiotic use compared with those who died (17 of 24 vs seven of 15 and 17 of 24 vs nine of 15, respectively). A total of 18 patients failed to achieve adequate source control within 7 days from BSI diagnosis. Four patients had persistent BSI despite 14 days of appropriate treatment.

Among patients who did not survive during hospital admission, higher incidences of acute kidney injury (53.3% vs 33.3%), ICU admission (20.0% vs 12.5%), severe sepsis (86.7% vs 45.8%), and septic shock (53.3% vs 25.0%) from day 1 to 7 of bacteremia were noted.

The all-cause in-hospital mortality rate was 38.5% (15 of 39; 95% confidence interval 23.4-55.4). Five deaths were attributed to Kp infection. The median time from BSI diagnosis to death was 9 days (interquartile range 34.0). Table 1 summarizes the demographic and clinical characteristics.

**Table 1:** Clinical and demographic characteristics of patients with Kp bloodstream infections (N = 39).

	Survivors (n = 24)	Non-survivors (n = 15)	*P*
Demographics			
Mean age; SD (years)	53.0; 15.8	55.1; 20.9	0.719
Male sex	12 (50.0%)	9 (57.1%)	0.542
Comorbidities			
Myocardial infarction	1 (4.2%)	2 (13.3%)	0.296
Congestive heart failure	2 (8.3%)	1 (6.7%)	0.849
Cerebrovascular disease	3 (12.5%)	2 (13.3%)	0.940
Chronic pulmonary disease	1 (4.7%)	0 (0.0%)	0.423
Diabetes without complications	8 (33.3%)	2 (13.3%)	0.164
Diabetes with end organ damage	5 (20.8%)	4 (26.7%)	0.674
Stroke with hemiplegia	1 (4.1%)	2 (13.3%)	0.296
Moderate or severe renal disease	2 (7.1%)	2 (14.3%)	0.617
Moderate or severe liver disease	0 (0.0%)	1 (6.7%)	0.200
Solid tumor, non-metastatic	3 (12.5%)	1 (6.7%)	0.559
Solid tumor, metastatic	4 (16.7%)	4 (26.7%)	0.452
Leukemia	3 (12.5%)	0 (0.0%)	0.154
Origin of infection			
Community-acquired	13 (54.2%)	7 (46.7%)	0.648
Hospital-acquired	11 (45.8%)	8 (53.3%)	
ICU admission at onset of bacteremia	2 (8.3%)	2 (13.3%)	0.617
Ventilator use at onset of bacteremia	5 (20.8%)	7 (46.7%)	0.089
Mean Pitt bacteremia score; SD	1.9; 2.7	5.5; 4.3	0.003
Presence of indwelling devices			
Central venous catheters	2 (8.3%)	4 (26.7%)	0.123
Urethral catheters	9 (37.5%)	7 (46.7%)	0.571
Ureteral stents/nephrostomies	4 (14.3%)	0 (0.0%)	0.095
Biliary stents	1 (4.2%)	3 (20.0%)	0.113
Gastrointestinal stents	0 (0.0%)	3 (20.0%)	0.023
Surgical procedures			
Colostomy	0 (0.0%)	2 (13.3%)	0.066
Choledochojejunostomy	0 (0.0%)	2 (13.3%)	0.066
Immunocompromised states			
Neutropenia within 30 days of anticancer therapy	3 (12.5%)	0 (0.0%)	0.154
Cellular immunodeficiency	1 (4.2%)	0 (0.0%)	0.423
Focus of infection			
Primary bacteremia, without documented source	2 (8.3%)	1 (6.7%)	0.849
Central venous catheter infection	2 (8.3%)	2 (13.3%)	0.617
Peripheral venous catheter infection	0 (0.0%)	1 (6.7%)	0.200
Pneumonia	5 (20.8%)	4 (26.7%)	0.674
Pyelonephritis	2 (8.3%)	2 (13.3%)	0.617
Catheter associated urinary tract infection	6 (25.0%)	1 (6.7%)	0.147
Cholangitis	3 (12.5%)	3 (20.0%)	0.528
Spontaneous bacterial peritonitis	1 (4.2%)	0 (0.0%)	0.423
Secondary peritonitis/intra-abdominal abscess	4 (16.7%)	3 (20.0%)	0.792
Cellulitis	1 (4.2%)	0 (0.0%)	0.423
Decubitus ulcer	1 (3.6%)	0 (0.0%)	0.423
Superficial/muscular surgical site infection	3 (12.5%)	0 (0.0%)	0.154
Management			
Use of appropriate empiric antibiotic	17 (70.8%)	7 (46.7%)	0.131
Use of appropriate definitive antibiotic	17 (70.8%)	9 (60.0%)	0.485
Source control achieved by day 7 of bacteremia			
No	13 (54.2%)	5 (33.3%)	0.440
Yes	4 (16.7%)	4 (26.7%)	
Absence of infected sites requiring source control	7 (29.2%)	6 (40.0%)	
Clinical complications			
Acute kidney injury by day 30 (RIFLE criteria)			
End-stage renal disease	3 (12.5%)	2 (13.3%)	0.509
Failure	0 (0.0%)	1 (6.7%)	
Injury	4 (16.7%)	3 (20.0%)	
Risk	1 (4.2%)	2 (13.3%)	
ICU admission during days 1-7 of bacteremia	3 (12.5%)	3 (20.0%)	0.528
Severe sepsis during days 1-7 of bacteremia	11 (45.8%)	13 (86.7%)	0.011
Septic shock during days 1-7 of bacteremia	6 (25.0%)	8 (53.3%)	0.070
Persistent bacteremia by day 14 of antibiotics	3 (14.3%)	1 (7.1%)	0.515

Abbreviations: ICU, intensive care unit; Kp, *Klebsiella pneumoniae*; RIFLE, risk, injury, failure, loss of kidney function, and end-stage kidney disease.

### Phenotypic resistance

AST results are shown in Figure 2. High resistance rates were observed for ampicillin-sulbactam (48.7%; 19 of 39), piperacillin-tazobactam (43.6%; 17 of 39), cefazolin (48.7%; 19 of 39), cefoxitin (38.5%; 15 of 39), ceftriaxone (41.0%; 16 of 39), ceftazidime (43.6%; 17 of 39), cefepime (35.9%; 14 of 39), aztreonam (41.0%; 16 of 39), imipenem (33.3%; 13 of 39), meropenem (33.3%; 13 of 39), ertapenem (33.3%, 13 of 39), amikacin (23.1%; nine of 39), gentamicin (28.2%; 11 of 39), tobramycin (35.9%; 14 of 39), ciprofloxacin (53.8%; 21 of 39), levofloxacin (41.0%; 16 of 39), and trimethoprim-sulfamethoxazole (43.6%; 17 of 39).

**Figure 2:**
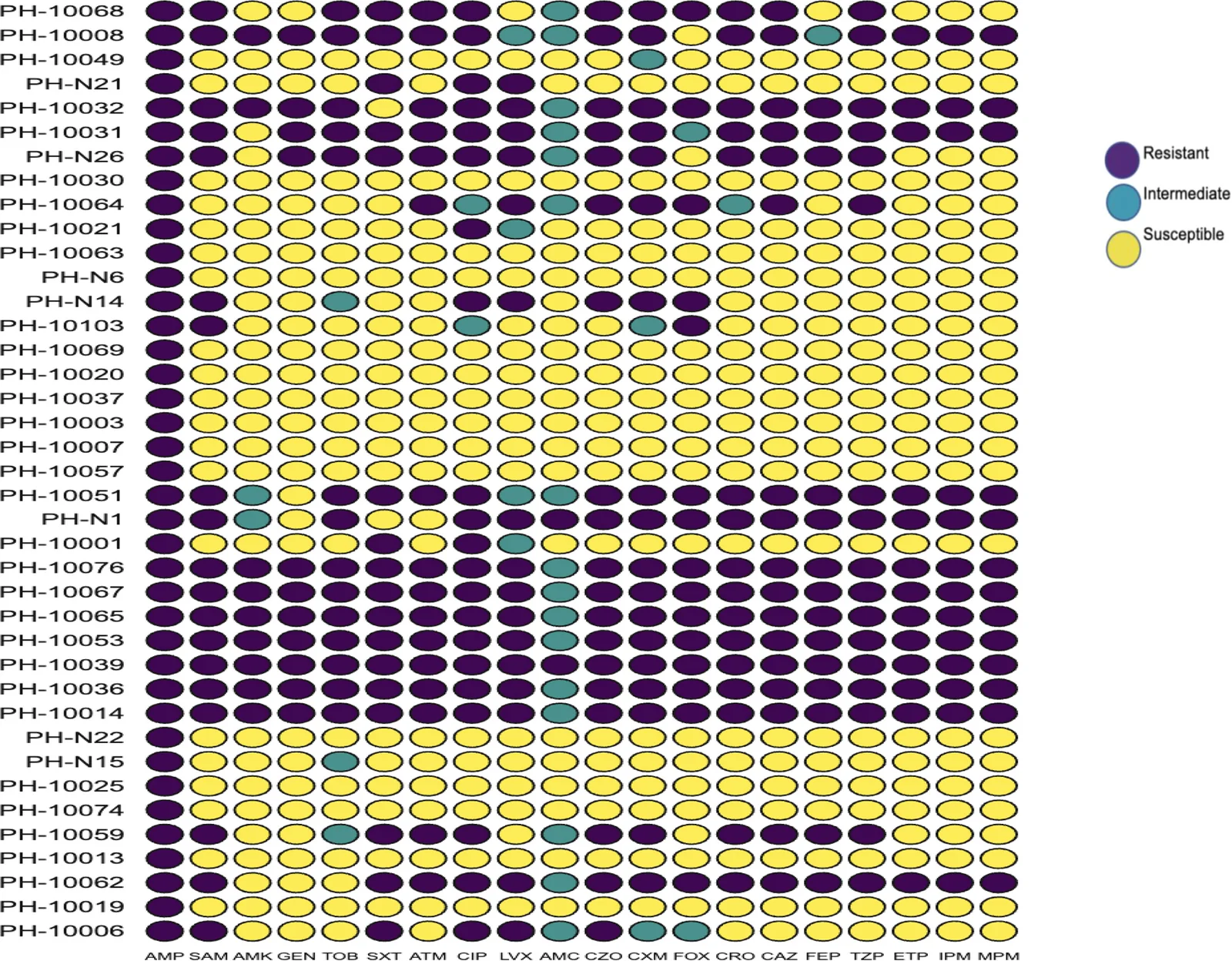
Phenotypic antimicrobial susceptibility of *Klebsiella pneumoniae* isolates. Created in BioRender. Poblete J. (2026) (https://BioRender.com/4kna2f0). Abbreviations: AMC, amoxicillin-clavulanic acid; AMK, amikacin; AMP, ampicillin; ATM, aztreonam; CAZ, ceftazidime; CIP, ciprofloxacin; CRO, ceftriaxone; CXM, cefuroxime; CZO, cefazolin; ETP, ertapenem; FEP, cefepime; FOX, cefoxitin; GEN, gentamicin; IPM, imipenem; LVX, levofloxacin; MPM, meropenem; SAM, ampicillin-sulbactam; SXT, trimethoprim-sulfamethoxazole; TOB, tobramycin; TZP, piperacillin-tazobactam.

Overall, 16 (41.0%) isolates were ESBL producers based on the phenotypic detection of ESBL and 13 (33.3%) were CR based on susceptibility testing [13].

### STs, genotypic resistance, and virulence profiles

WGS revealed ST437 as the most common sequence type (seven of 39; 17.9%), followed by ST23 (six of 39; 15.4%), ST101 (three of 39; 7.7%), and ST17 (three of 39; 7.7%). Capsular genotyping identified K36 as the most common type (seven of 39; 17.9%), followed by K1 (six of 39; 15.4%) and K17 (four of 39; 10.2%); only one K2 isolate was detected. The predominant O antigen genotype was O1ab (19 of 39; 48.7%), followed by O4 (eight of 39; 20.5%) and O2afg (five of 39; 12.8%). All ST23 isolates exhibited the K1:O1ab genotype combination, whereas all ST437 isolates were K36:O4 genotype.

Genotypic resistance was largely concordant with phenotypic data. *bla*_SHV-11_, encoding a non-ESBL β-lactamase, was the most frequent AMR gene (24 of 39). All isolates harbored β-lactamase genes, including 15 with *bla*_TEM-1_, six of which co-carried additional β-lactamase genes.

A total of 16 isolates were ESBL-positive, all harboring *bla*_CTX-M-15_. Carbapenemase genes were present in 13 isolates, including two *bla*_NDM-5_, two *bla*_NDM-1_, one *bla*_NDM-7_, two *bla*_KPC-2_, one *bla*_OXA-232_, four *bla*_NDM-5_ + *bla*_OXA-232_, and one *bla*_NDM-1_ + *bla*_KPC-2_.

Hypervirulence-associated genes identified included *rmpA* (10 of 39), *rmpA2* (10 of 39), *iuc* (11 of 39), and *iro* (10 of 39). A total of 10 (25.6%) isolates met the criteria for hvKp; five of these were ST23. One ST23 isolate was identified as CR-hvKp (designated PH-N1), which harbored *bla*_NDM-7_.

Plasmid-mediated fluoroquinolone resistance genes were found in 13 isolates, most commonly *qnrS* (eight of 13).

Figure 3 summarizes the distributions of STs, AMR genes, and virulence genes.

**Figure 3:**
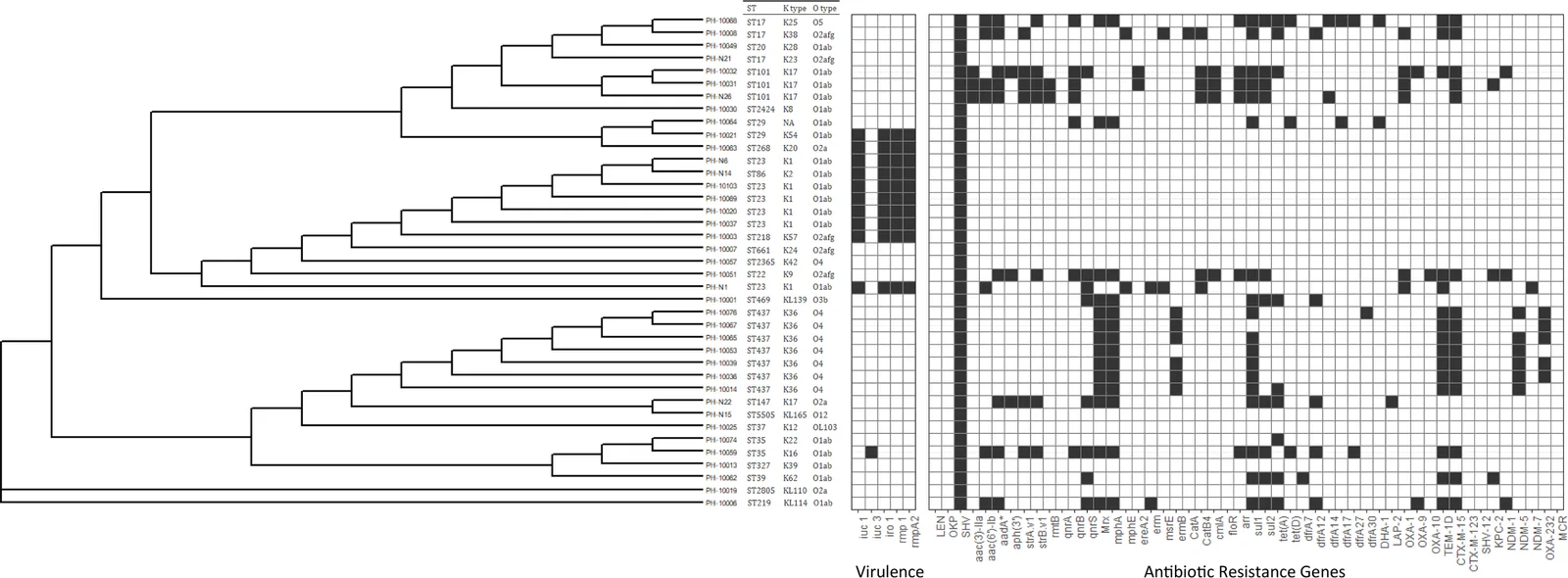
STs, genotypic antimicrobial susceptibility, and presence of hypervirulence genes in *Klebsiella pneumoniae* isolates. Abbreviation: ST, sequence type.

### Infection origin and patient outcomes stratified by hypervirulence genotypes

Table 2 shows the infection origins and outcomes of patients with hvKp and cKp BSI. Among 10 hvKp cases, nine were considered community-acquired and three in-hospital deaths were recorded (two hvKp and one CR-hvKp). The all-cause in-hospital mortality rate (41.4% vs 30.0%, *P* = 0.24), ICU admission rate (20.7% vs 0.0%, *P* = 0.118), and development of severe sepsis (68.9% vs 40.0%, *P* = 0.104) during days 1-7 of bacteremia were numerically higher in the cKp group. In contrast, progression to septic shock (40.0% vs 34.5%, *P* = 0.754) and the proportions of deaths attributed to Kp infection (66.7% vs 25.0%, *P* = 0.245) were more common in the hvKp group. The origin of infection between the two groups was the only variable that reached statistical significance (*P* = 0.005).

**Table 2:** Infection origin and outcomes of patients with hvKp and cKp infections (N = 39).

	hvKp (n = 10)	cKp (n = 29)	*P*
Infection origin			
Community-acquired	9 (90.0%)	11 (37.9%)	0.005
Hospital-acquired	1 (10.0%)	18 (62.1%)	
Outcomes			
All-cause in-hospital mortality	3 (30.0%)	12 (41.4%)	0.524
Mortality attributed to Kp	2 of 3 (66.7%)	3 of 12 (25.0%)	0.245
Intensive care unit admission during days 1-7 of bacteremia	0 (0.0%)	6 (20.7%)	0.118
Severe sepsis during days 1-7 of bacteremia	4 (40.0%)	20 (68.9%)	0.104
Septic shock during days 1-7 of bacteremia	4 (40.0%)	10 (34.5%)	0.754
Persistent bacteremia by day 14 of antibiotics	2 (20.0%)	2 (6.9%)	0.313

Abbreviations: cKp, classic *Klebsiella pneumoniae*; hvKp, hypervirulent *Klebsiella pneumoniae*; Kp, *Klebsiella pneumoniae*.

## Discussion

Kp infections have a global distribution, with a particularly high prevalence in East and Southeast Asia, Northern Africa, and Southern Europe [2]. In the Philippines, however, clinical and molecular data remain scarce despite the high burden of disease.

In our cohort, Kp BSIs were more common among males and middle-aged adults, differing from a study that showed a higher prevalence among older patients, without sex predilection [17]. Male sex has nonetheless been associated with MDR Kp infections in some studies [18,19]. Diabetes mellitus and solid tumor malignancies were the most frequent comorbidities, consistent with findings from other countries in the region [[17], [18], [19]]; both conditions are known to predispose to infection. Diabetes mellitus impairs neutrophil and granulocyte function, alters cytokine production, and enhances capsule formation, whereas malignancy increases infection risk through barrier disruption, tumor- and therapy-related immunosuppression, and exposure to invasive procedures [20].

We observed no major difference in frequency between hospital-acquired and community-acquired infections, aligning with recent genomic surveys in the Philippines [7]. National ARSP data, however, report slightly fewer hospital-acquired infections [7]. Notably, intra-abdominal infections were the most common focus in our cohort, as in earlier studies from Taiwan [17]. However, pneumonia was the most common diagnosis linked to bacteremia. Recent studies have reported an increasing frequency of urinary tract and respiratory tract infections as predominant sources [18,19]. These shifts may reflect the global spread of MDR and hvKp across community and hospital settings [21,22].

The all-cause in-hospital mortality (38.5%) in our study was higher than that reported in other cohorts (23%-31%) [17,18]. This may be related to the severity of illness at BSI onset, reflected by the frequent need for mechanical ventilation, higher Pitt bacteremia scores, and higher rates of severe sepsis and septic shock among non-survivors. The predominance of pneumonia may also contribute because it is a recognized risk factor for mortality (adjusted odds ratio 4.14; 95% confidence interval 2.3-7.6) [17]. In addition, the lower frequency of appropriate antibiotic use in the non-surviving group is an important variable affecting mortality. In the Philippines, meropenem combined with polymyxin B is often used as an alternative treatment for CR Kp when ceftazidime-avibactam and aztreonam are not available. This is considered an inappropriate treatment regimen due to a lack of evidence of efficacy [16]. Other factors likely influencing poor outcomes include comorbidities, indwelling devices, previous surgeries, prolonged antibiotic use, delayed source control, and delays in presentation or treatment initiation. Our limited sample size, however, precludes regression analysis to identify independent predictors of mortality.

AMR remains a major public health challenge, particularly in low- to middle-income countries, where antibiotic use is less regulated, diagnostics are limited, and surveillance is weak [3]. In our study, the rates of ESBL (41.0%) and carbapenem resistance (33.3%) were alarmingly high. Although the ARSP 2024 data report similar ESBL prevalence (46.9%-51.6%), carbapenem resistance rates were notably lower [7]. By contrast, studies from Korea (40% ESBL, 23% carbapenem resistance) [23] and Saudi Arabia (34.9% ESBL, 36.2% carbapenem resistance) [18] reported lower resistance rates. Despite regional variation, the global trend clearly shows an increasing MDR Kp prevalence [24,25]. If left unaddressed, this will have devastating consequences for health systems and economies.

The re-emergence of polymyxins (polymyxin B and colistin) as a last-resort therapy against MDR and extensively drug-resistant Gram-negative bacilli has been undermined by misuse and the subsequent emergence of resistance [7]. The ARSP 2024 reports CR Kp harboring the plasmid-mediated polymyxin resistance (*mcr-1*) gene, causing modification in the cell membrane, reducing antibiotic activity [7]. In our study, none of the isolates harbored *mcr-1*. Given the limited number of isolates analyzed, our results should be interpreted with caution and may not reflect the ARSP 2024 prevalence (6.7%) [7] or the pooled prevalence of 3.1% [1].

WGS revealed ST437 and ST23 as the most common clones. The predominance of ST437 likely reflects its nosocomial association. ST437 is a globally disseminated clone that is frequently linked to health care–associated infections and known to carry carbapenemase and other AMR genes [26]. In contrast, ST23 is classically associated with hvKp and disseminated infections [27]. Consistent with this, all ST23 strains in our study had the K1:O1ab genotype. A previous Philippine survey reported ST147 and ST39 as dominant clones [7], underscoring regional variability. Multiple co-existing AMR genes were identified, with *bla*_CTX-M_ and *bla*_SHV_ as the most common ESBL genes and *bla*_NDM_ and *bla*_KPC_ as the most common carbapenemase genes, consistent with global reports [27,28]. These resistance determinants, combined with limited access to molecular diagnostics and effective therapies, likely contributed to the high mortality in our cohort, as seen elsewhere [29].

Importantly, to the best of our knowledge, this is the first local study to investigate hypervirulence genes in Kp BSIs. We identified 10 hvKp isolates, nine of which showed no major phenotypic or genotypic resistance, consistent with the evolutionary separation of virulence and resistance plasmids [11]. However, one isolate was classified as CR-hvKp, harboring *bla*_NDM-7_. Mortality among our hvKp cases was high (three of 10; 30%), although this lower than what was reported in India (56.2%) [30]. These findings emphasize the need for genomic surveillance to detect AMR and virulence determinants, enabling early recognition of high-risk infections and guiding timely interventions.

This study has several limitations. First, being a single-center study, the findings may not represent the broader epidemiology of Kp in the Philippines. Molecular characteristics may vary across institutions. Second, some confounding factors that influence outcomes, such as delays in admission, diagnostic availability, and treatment timeliness, were not captured. Third, susceptibility to colistin and tigecycline was not routinely tested. This prevented full characterization of resistance phenotypes and identification of pan-resistant Kp isolates. Finally, the relatively small sample size precluded multivariable analysis of predictors of mortality, and the study results should be interpreted cautiously.

## Conclusion

Kp is a significant cause of BSI among Filipino patients, particularly those with comorbidities, immunocompromised states, or previous surgical interventions. The high prevalence of ESBL-producing and CR strains underscores the urgent threat posed by AMR in this setting. The detection of hvKp and CR-hvKp is a testament to the global spread of this critical priority pathogen. A sustained national genomic surveillance is essential to monitor the evolving clinical and molecular epidemiology of Kp and inform timely infection control and treatment strategies.

## Funding

This study is part of the Deciphering the AMR and Hypervirulence in Klebsiella pneumoniae infections (DETAIIL-KP), a collaborative project under the East Asia Joint Research Program (e-ASIA JRP). The National Institutes of Health-University of the Philippines Manila received funding from the Philippine Council for Health Research and Development (PCHRD). Fujita Health University received funding from the Japan Agency for Medical Research and Development (AMED). Washington University in St. Louis received funding from the Civilian Research and Development Foundation (CRDF) Global.

## Ethical approval

This study received approval from the UPMREB, with protocol code 2023-0210-01.

## CRediT authorship contribution statement

**Christian Francisco:** Conceptualization, Data curation, Formal analysis, Funding acquisition, Investigation, Methodology, Project administration, Resources, Software, Supervision, Validation, Visualization, Writing – original draft, Writing – review & editing. **Angelo dela Tonga:** Conceptualization, Data curation, Formal analysis, Investigation, Methodology, Project administration, Software, Validation, Writing – original draft, Writing – review & editing. **Stessi Marie Geganzo:** Data curation, Formal analysis, Investigation, Methodology, Project administration, Software, Supervision, Validation, Writing – original draft, Writing – review & editing. **Joana Marie Gantuangco:** Data curation, Formal analysis, Investigation, Methodology, Project administration, Validation, Writing – original draft, Writing – review & editing. **Jonnel Poblete:** Data curation, Formal analysis, Investigation, Methodology, Project administration, Validation, Writing – original draft, Writing – review & editing. **Ia Marie Cruz:** Data curation, Formal analysis, Investigation, Methodology, Project administration, Validation, Writing – original draft, Writing – review & editing. **Bryan Santiago:** Data curation, Formal analysis, Investigation, Methodology, Software, Validation, Writing – original draft, Writing – review & editing. **Janet S. Lee:** Conceptualization, Data curation, Formal analysis, Funding acquisition, Investigation, Methodology, Project administration, Resources, Supervision, Validation, Writing – original draft, Writing – review & editing. **Tatsunori Oyabu:** Data curation, Formal analysis, Investigation, Methodology, Project administration, Software, Validation, Writing – original draft, Writing – review & editing. **Masahiro Suzuki:** Conceptualization, Data curation, Formal analysis, Investigation, Methodology, Project administration, Resources, Software, Supervision, Validation, Writing – original draft, Writing – review & editing. **Sohei Harada:** Conceptualization, Data curation, Formal analysis, Funding acquisition, Investigation, Methodology, Project administration, Resources, Software, Supervision, Validation, Writing – original draft, Writing – review & editing. **Yohei Doi:** Conceptualization, Data curation, Formal analysis, Funding acquisition, Investigation, Methodology, Project administration, Resources, Software, Supervision, Validation, Writing – original draft, Writing – review & editing.

## Declaration of competing interest

CF received honoraria for lectures from bioMérieux, Bioneer, Biocare Lifesciences Inc., and Unilab Inc. SH received honoraria for lectures and manuscript writing from Shionogi, MSD Pfizer, Denka, Ushio Inc., and Eiken Chemical Co., Ltd. outside the submitted work. YD received speaking fees from Shionogi, Gilead, BD, and Chugai and served as a consultant for GSK, Roche, and Meiji Seika Pharma. The remaining authors have no competing interests to declare.

## Appendix Supplementary materials

Image, application 1
