# Supplementary material for: Clinical and molecular profile of Klebsiella pneumoniae bloodstream infections in a national referral hospital in the Philippines
Source: IJID Reg. 2026 Oct 15;21:101039. doi: 10.1016/j.ijregi.2026.101039 (PMC13742235; doi:10.1016/j.ijregi.2026.101039)
Supplement: Supplementary file 1 [file mmc1.docx]

Supplementary Table: Antibiotic regimen given as empiric and definitive treatment in patients with *Klebsiella pneumoniae* (Kp) bloodstream infections

| Empiric Treatment | | |
| --- | --- | --- |
| Antibiotic Regimen, frequency | Survivors | Non survivors |
| Meropenem | 7 | 2 |
| Ceftriaxone | 3 | 0 |
| Ciprofloxacin | 2 | 0 |
| Levofloxacin | 2 | 0 |
| Piperacillin Tazobactam | 2 | 1 |
| Meropenem + Polymyxin B | 2 | 2 |
| Ampicillin Sulbactam | 1 | 0 |
| Cefepime | 1 | 0 |
| Ceftazidime + Azithromycin | 1 | 2 |
| Cefazolin | 1 | 0 |
| Ertapenem | 1 | 1 |
| Co-amoxiclav | 1 | 0 |
| Ceftriaxone + Metronidazole | 1 | 0 |
| Meropenem + Amikacin | 1 | 3 |
| Cefepime + Metronidazole | 1 | 0 |
| Meropenem + Levofloxacin | 1 | 1 |
| Ceftazidime -Avibactam | 0 | 1 |
| Ceftriaxone + Levofloxacin + Metronidazole | 0 | 1 |
| Cefoxitin | 0 | 1 |
| Piperacillin Tazobactam + Levofloxacin | 0 | 1 |
| Definitive Treatment | | |
| Antibiotic Regimen | Survivors | Non survivors |
| Ceftriaxone | 6 | 0 |
| Ertapenem | 3 | 0 |
| Ciprofloxacin | 2 | 0 |
| Meropenem | 2 | 0 |
| Meropenem + Amikacin | 2 | 2 |
| Meropenem + Polymyxin B | 2 | 3 |
| Meropenem + Polymyxin B+ Doxycycline | 2 | 0 |
| Ceftazidime Avibactam | 1 | 4 |
| Ceftazidime Avibactam + Aztreonam | 1 | 0 |
| Levofloxacin | 1 | 0 |
| Ceftriaxone + Metronidazole | 1 | 0 |
| Cefepime | 1 | 0 |
| Meropenem + Levofloxacin | 1 | 1 |
| Ceftazidime + Azithromycin | 0 | 2 |
| Piperacillin Tazobactam | 0 | 2 |
| Piperacillin + Tazobactam + Levofloxacin | 0 | 1 |
